# Sanbo Scoring System, Based on Age and Pre-treatment Hematological Markers, is a Non-invasive and Independent Prognostic Predictor for Patients with Primary Glioblastomas: A Retrospective Multicenter Study

**DOI:** 10.7150/jca.33047

**Published:** 2019-09-07

**Authors:** Peng-fei Wang, Jianbin Zhang, Hong-qing Cai, Zhe Meng, Chun-jiang Yu, Shou-wei Li, Jing-hai Wan, Chang-Xiang Yan

**Affiliations:** 1 Department of Neurosurgery, Sanbo Brain Hospital, Capital Medical University, China; 2 Department of Neurosurgery, National Cancer Center/Cancer Hospital, Chinese Academy of Medical Sciences and Peking Union Medical College, China

**Keywords:** Glioblastoma, Prognostic factor, Hematological markers, Inflammation, Nutrition

## Abstract

Various hematological markers are associated with survival in patients with glioblastomas (GBMs), as they reflect inflammation and nutrition status. However, single markers are insufficient for predicting prognosis in GBM, and a comprehensive scoring system is needed. In this study, we developed a simple, inexpensive, and non-invasive scoring system, referred to as the Sanbo Scoring System (SSS), to predict survival in patients with GBMs. Patients with GBM were retrospectively assigned to two independent cohorts at Sanbo Brain Hospital and National Cancer Center/Cancer Hospital. Clinical records, including age, routine blood tests, biochemistry and coagulation examinations, and IDH-1 status, were collected. In total, 274 and 87 patients with GBMs at Sanbo Brain Hospital and National Cancer Center/Cancer Hospital were included as derivation and validation cohorts, retrospectively. We developed the SSS based on data for the derivation cohort, i.e., age, neutrophil-to-lymphocyte ratio (NLR), platelet-to-lymphocyte ratio (PLR), albumin-to-globulin ratio (AGR), and fibrinogen levels. These patients were divided into three groups that differed with respect to age, inflammation-nutrition status, and overall survival (p < 0.001), i.e., SSS 0, 1, and 2. NLR, PLR, and fibrinogen levels were lower and AGR was higher in the SSS 2 group than in the other groups, indicating better inflammation and nutrition statuses. Additionally, the longest overall survival was observed in this group. A multivariate analysis showed that SSS was an independent prognostic factor. The validation cohort supported all the results. SSS was a simple, non-invasive, and effective scoring system, and independently predicted survival in GBMs.

## Introduction

Glioblastomas (GBMs) are the second most common central nervous system tumors and have a poor prognosis 1. Classification of GBMs is highly important for predicting survival and optimizing therapeutic strategies 2. Pathology is the only robust and informative method for classification to date, but is invasive and requires a biopsy, at minimum. Plasma cell-free circulating tumor DNA detection is effective for many cancers, but is nearly undetectable in patients with gliomas due to the brain-blood-barrier 3. Although cerebrospinal fluid is ideal for detecting circulating tumor DNA 4, this method is expensive and technologically demanding. A simple, low-cost, and non-invasive method for classification is needed, especially for patients with newly diagnosed GBMs.

Our results and those of other previous studies have identified several prognostic factors in GBMs, such as the neutrophil-to-lymphocyte ratio (NLR) 5-7, platelet-to-lymphocyte ratio (PLR) 5, 6, albumin-to-globulin ratio (AGR) 8, prognostic nutrition index (PNI) 8, and albumin 9. These hematological markers reflect the nutritional and inflammatory status in GBMs. Furthermore, combinations of these hematological markers, such as fibrinogen (FIB) and albumin, could better stratify gliomas 10. These results provide the basis for the development of a scoring system that comprehensively integrates these inflammation and nutrition makers. However, the cutoff values for these prognostic factors, such as NLR 5-7, are not uniform, and it is therefore difficult to apply these factors in clinical practice. In this study, we developed a new scoring system, named the Sanbo Scoring System (SSS), incorporating age, NLR, PLR, AGR, and fibrinogen; this system could be effective for prognostic evaluations of patients with GBMs. To validate our results for SSS, we used clinical data from the National Cancer Center/Cancer Hospital, Chinese Academy of Medical Sciences and Peking Union Medical College. We also set fixed cutoff values for age and hematological markers, with the goal of developing a universal scoring system for application in clinical practice.

## Patients and Methods

### Patients

Patients with GBMs from the derivation cohort and validation cohort were recruited from the Department of Neurosurgery of Sanbo Brain Hospital and National Cancer Center/Cancer Hospital, and the Chinese Academy of Medical Sciences and Peking Union Medical College, respectively. All the patients had a chemoradiotherapy according to Stupp's protocols 11. Inclusion criteria were as follows: Patients were first diagnosed of GBMs by pathology 2. Patients complete routine blood test results, blood biochemical and coagulation results before any treatment. Patients underwent a surgery or at least biopsy with the examination of IDH-1^R132H^ mutations. Exclusion criteria were as follows: Patients suffered from other coexisting diseases, including infection, hematological disorders, autoimmune diseases, renal or hepatic dysfunction. Patients diagnosed with other malignancies. Patients had received preoperatively medical treatment, including glucocorticoid treatment, radiation, and chemotherapy. Written informed consent forms were obtained from patients for sampling and research. The Ethics Committees approved of this study for both the derivation (No. SBNK-2018-003-01) and validation cohort (No. NCC2014G-12).

### Blood examination and data collection

Peripheral venous blood samples were obtained before surgery and then conveyed to the Clinical Laboratory Department within 30 min for routine tests. Complete blood counts, blood biochemical indexes, and coagulation parameters were collected for further analyses. In this study, NLR was defined as the ratio of the absolute neutrophil count to the absolute lymphocyte count, PLR was defined as the absolute platelet count divided by the absolute lymphocyte count, and AGR was calculated as the ratio of serum albumin to globulin.

### Immunohistochemistry

The procedures of IHC was performed as we previously described 12. The detection of IDH-1^R132H^ mutations by immunohistochemistry was performed as described previously using an anti-IDH-1^R132H^ antibody (working solution, ZM0447, ZSGB-BIO, Beijing, China) as a primary antibody. A cut-off value of 10% was used to evaluate the presence or absence of IDH-1^R132H^.

### Statistical analysis

All data were analyzed using SPSS 22.0 (IMB Inc., Armonk, New York). Differences in continuous variables between groups were analyzed by unpaired *t*-tests or a variance analysis, and discrete variables were analyzed by chi-square tests. The Kaplan-Meier method was used to draw an overall survival (OS) curve. A univariate Cox analysis was used to analyze the prognostic significance of variables. A multivariate analysis by the Cox proportional-hazard model was used to determine independent prognostic factors. A two-sided p-value of <0.05 was considered statistically significant.

## Results

### Patient demographic properties

There were 274 patients in the derivation cohort and 87 patients in the validation cohort. All patients included in the study had pretreatment hematological examinations and survival data. Baseline clinicopathological parameters in the two cohorts were highly similar, including age, gender, NLR, PLR, AGR, FIB, IDH-1 mutations, GTR, chemoradiotherapy, follow-up period, and OS (Table 1).

### Development of SSS

**Figure 1:**
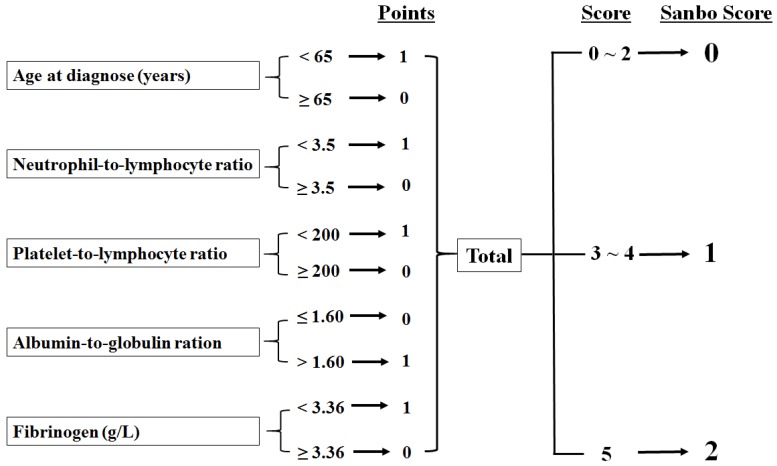
Calculation of the Sanbo Brain prognostic score

SSS was determined by the following five parameters: age at diagnosis (normal, <65 years), NLR (normal, <3.5), PLR (normal, <200), AGR (normal, >1.6), and fibrinogen (normal, <3.36). We divided patients into three groups: those with normal values for five parameters were classified as SSS 2, patients with normal values for three or four parameters were assigned to SSS 1, and others were assigned to SSS 0 (Fig. 1).

### Association between SSS and clinicopathological factors

According to our scoring system, age, NLR, PLR, AGR, and fibrinogen levels were significantly greater in SSS 2 than in SSS 0 and 1 (Table 2). The incidence of IDH-1 mutations did not differ among the three groups in the derivation cohort (p = 0.638) or the validation cohort (p = 0.050). However, there were more males in SSS 1 and 2 than in SSS 0 in the derivation cohort (p = 0.007). This difference was not observed in the validation cohort (p = 0.850).

### Survival analysis

The median follow-up was 12.77 (3.80-48.97) months in the derivation cohort. In total, 186 (67.88%) patients died due to tumor recurrence at the last follow-up, with 50.2% and 13.1% 1- and 2-year survival rates, respectively. The results described above were highly consistent with the results obtained in the validation group. A total of 55 (63.2%) patients died at the last follow-up, and 1- and 2-year survival rates were 57.3% and 14.5%, respectively.

**Figure 2:**
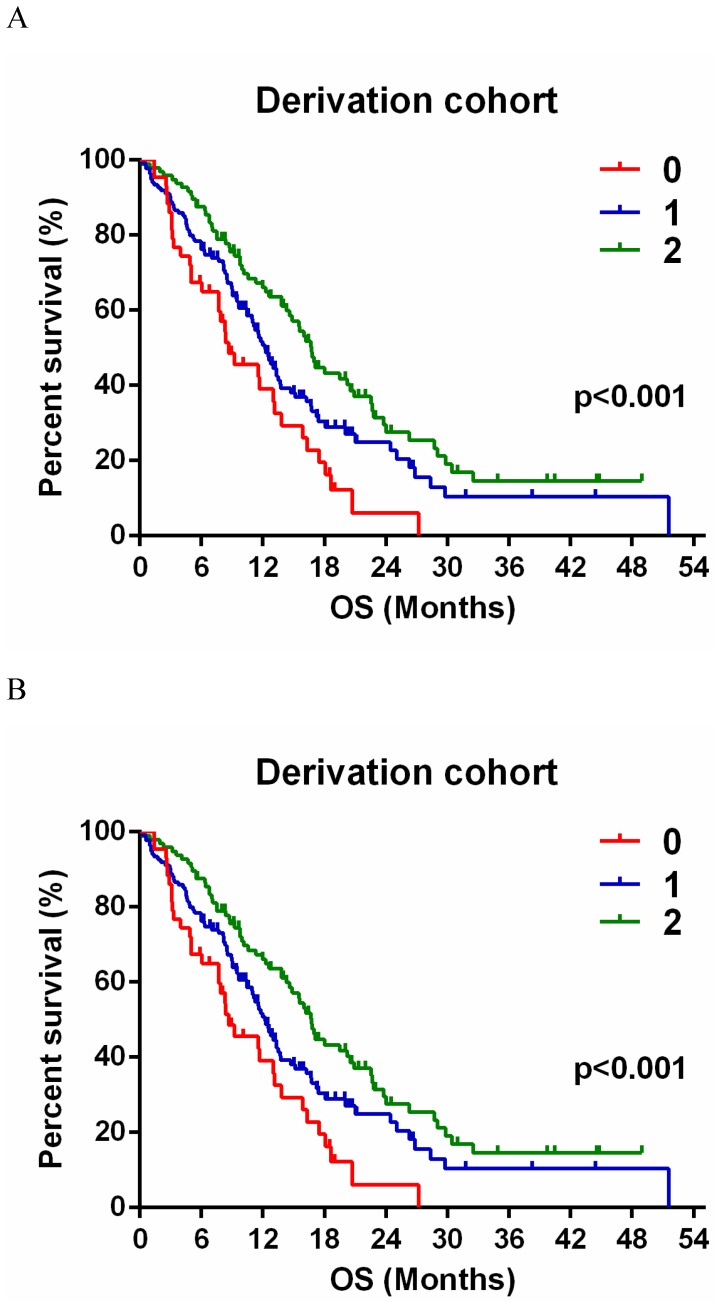
** A**, Kaplan-Meier survival curve for patients with GBMs according to SSS group in the derivation group. SSS = 0, n = 43; SSS = 1, n = 135; SSS = 2, n = 96. **B** Survival curve of SSS in validation group. SSS = 0, n = 19; SSS = 1, n = 45; SSS = 2, n = 23.

The estimated relative risk (RR) of death was 64.9% lower in SSS 1 and 74.7% lower in SSS 2 than that in SSS 0 (Table 3). Additionally, the RR was significantly lower by 83.3% (70.6-98.0%) in SSS 2 than in SSS 1 (Fig. 2A). In the validation group, the estimated RR was significantly lower, by 65.9% and 69.9%, in SSS 1 and 2 respectively, than in SSS 0 (Table 3). RR was 72.0% (50.5-102.6%) lower in SSS 2 than in SSS 1, but this difference was not significant (p = 0.069, Fig. 2B).

In a univariate analysis, we found that SSS, IDH-1^R132H^ mutations, gross total resection, and complete chemoradiotherapy were significantly associated with a favorable clinical outcome in the derivation group. There was a difference in the validation group, but this difference was small (Table 3). However, a multivariate analysis of the two independent cohorts both showed that SSS, IDH-1^R132H^ mutations and chemoradiotherapy were independent prognostic factors.

## Discussion

In this study, we developed and validated, internally and externally, a scoring system for evaluating RR in patients with GBMs. This system, referred to as SSS, showed independent prognostic value with unified cutoff values for continuous variables. The SSS reflected a combined state of nutrition, inflammation, and coagulation in GBMs. Thus, our system can be used to non-invasively and effectively identify patients with a high risk of a shorter OS.

The prognostic significance of hematological markers has recently been established in a variety of cancers. NLR is the most common prognostic marker in GBMs, and its cutoff values range from 4-7 5-7. Various cutoff values have also been used for the PLR 5, 6, PNI 8, 13 and red blood cell distribution width 14, 15. The different cutoff values for these makers can be explained by study heterogeneity, including differences in age, IDH mutations, surgery, and chemoradiotherapy. These results have shown that a single marker is not sufficient to predict survival in patients with GBMs. Additional markers could reflect inflammation, nutrition, and coagulation states simultaneously. For example, a scoring system developed by He et al.^10^ based on the combination of plasma fibrinogen and albumin can predict progression-free survival and OS in patients with high-grade gliomas. Another prognostic score simultaneously considering NLR, the lymphocyte-to-monocyte ratio, albumin, and cholesterol is a simple and effective tool for predicting survival in patients with colorectal cancers 16.

One benefit of our scoring system is that it accounts for age, which is a powerful prognostic factor according to most studies of GBMs 10, 14. Additionally, SSS was developed based on the nutritional and inflammatory status. The prognostic value of this scoring system was independent of gender, IDH status, surgical resection, and chemoradiotherapy. SSS could be widely applied owing to its unified cutoff values and independent prognostic values.

The components of SSS were age, neutrophils, platelets, lymphocytes, albumin, globulin, and fibrinogen. A younger age was associated with favorable genetic changes, such as IDH mutation and ATRX loss, which were associated with a better clinical outcome in patients with GBMs 17, 18. Additionally, the cutoff value of 65 years was consistent with that in a previous report 19. Circulating neutrophils promoted tumor progression by secreting arginase I for immunosuppression and vascular endothelial growth factor for angiogenesis 20. Blood platelets migrate into the tumor environment and facilitate cancer cell metastasis, invasion, and immune suppression 21. Lymphocytes protect against tumor progression and prolong OS in patients with GBMs 22, 23. Consequently, higher NLR and PLR values indicated stronger immune suppression and were associated with a worse OS in GBMs. Lower levels of albumin were induced by tumor necrosis factor (TNF)-α, Interleukin (IL)-1β, and IL-6 24, and the latter cytokines inhibited T effector cells in GBMs 25. Globulin was another systemic inflammation marker, and a lower AGR indicated a systemic inflammatory response 26. Albumin was a marker of nutrition status. There is an active interaction between inflammation and nutrition in cancer 27. In patients with GBMs, an abnormal body mass index (BMI, kg/m^2^) indicating a patient is underweight (BMI < 18.5 kg/m^2^) or overweight (BMI ≥ 30 kg/m^2^) is associated with a worse OS 28. Moreover, cholesterol is a positive prognostic marker, with a crucial role in the nutrition status in GBMs 29. Plasma fibrinogen suppresses anti-tumor immunity by activating neutrophils 20, 30 and inhibiting natural killer cells 31. Taken together, the components of SSS were indicators of inflammation, immune-suppression, and nutrition status in GBMs.

Our study had several limitations. It was a retrospective study, suggesting the potential for a high recall bias. The external validation was an efficient strategy to account for this limitation. However, future prospective and multicenter studies are needed to confirm these results. Secondly, there were more patients in the derivation cohort than in the validation cohort, and this sample size difference might result in differences in results. For example, we observed a gender difference among the SSS groups, but the same results were not obtained in the validation cohort. Inconsistent NLR results between genders have been reported in GBMs 15, 32. The scoring system developed by He et al. showed no gender difference in GBMs 10. Similarly, gender was not a prognostic factor in our study. Accordingly, the inflammation status with respect to gender should be investigated further. Thirdly, the red blood cell distribution width, PNI, albumin, and blood lipids were not included in the SSS. Combinations of these hematological markers might produce a better scoring system. Multidisciplinary approaches, including approaches by researchers in mathematics, clinical laboratories, and medical fields, are needed to create the optimal system. Fourthly, these inflammation markers have diagnostic value for glioma. NLR is positively correlated with glioma grade with a cutoff of 2.59 32. Moreover, combinations of these markers, such as NLR and the lymphocyte-monocyte ratio, predict glioma grade and diagnosis with a high sensitivity and specificity 33. Furthermore, a reduced NLR during concurrent chemoradiotherapy is associated with an improved OS in patients with GBMs 34. A decreased NLR also predicts a better progression-free survival in patients with advanced cancers treated with PD-1 inhibitors 35. These results suggest that SSS or another scoring system should be used to determine treatment strategies and may provide additional prognostic value. Lastly, we admitted that there are limitations in regards to the development and statistical analysis of the scoring system. But this scoring showed that combination of these markers together could yield a prognostic indicator in glioblastoma, and encourage future studies to include these factors when building a predictive scoring system.

## Conclusions

Pretreatment SSS was an easy and effective scoring system and provided independent prognostic value for patients with GBMs. A low SSS was associated with strong immune suppression and a poor nutrition status. Our results were validated using an independent cohort. This novel system may improve GBM classification, and its effectiveness and diagnostic and prognostic value should be further evaluated.

## Figures and Tables

**Table 1:** Baseline Characteristics

	Derivation cohort (n = 274)	Validation cohort (n = 87)	*P* value
Age (Median, range, years)	54 (15-80)	57 (15-80)	0.641
Women (%)	111 (40.51%)	40 (45.98)	0.368
NLR	3.48 ± 2.87	4.16 ± 3.38	0.067
PLR	150.04 ± 77.38	168.14 ± 93.18	0.072
AGR	1.74 ± 0.36	1.75 ± 0.30	0.863
FIB (g/L)	2.67 ± 0.68	2.84 ± 0.78	0.059
IDH-1 mutation	42 (15.33)	18 (20.69)	0.355
GTR (%)	189 (68.98)	56 (64.37)	0.422
Complete chemoradiotherapy	115 (41.97)	45 (51.72)	0.111
Follow-up period (Median, months)	12.77 (3.80-48.97)	15.23 (2.43-45.97)	0.583
Overall survival (Median, months)	13.13 (11.42 ± 14.84)	15.37 (9.77 ± 20.97)	0.312

^#^ The data of NLR, PLR, AGR and FIB was presented as Mean ± SD.

**Table 2:** Association of SSS with clinicopathological factors

Variables	Derivation cohort			Validation cohort		
	SSS 0 (n = 43)	SSS 1 (n = 135)	SSS 2 (n = 96)	SSS 0 (n = 19)	SSS 1 (n = 45)	SSS 2 (n = 23)
Age (Mean ± SD)	58.65 ± 13.35	54.34 ± 13.07	46.86 ± 12.00^***###^	63.21 ± 9.32	53.73 ± 13.53^*^	43.83 ± 14.52^***#^
Gender (F/M)	26 / 17	54 / 81	31 / 65	8 / 11	22 / 23	10 /13
NLR	6.94 ± 4.42	3.44 ± 2.21^***^	1.99 ± 0.62^***###^	7.27 ± 4.61	3.88 ± 2.66^***^	2.13 ± 0.53^***^
PLR	236.28 ± 105.30	149.13 ± 67.63^***^	112.70 ± 32.80^***###^	268.77 ± 100.85	151.00 ± 80.29^***^	118.53 ± 28.41^***^
AGR	1.46 ± 0.24	1.64 ± 0.29^**^	2.00 ± 0.32^***###^	1.61 ± 0.35	1.74 ± 0.30	1.87 ± 0.21^*^
FIB (g/L)	3.20 ± 0.75	2.71 ± 0.69^***^	2.38 ± 0.46^***###^	3.50 ± 0.83	2.81 ± 0.69^***^	2.33 ± 0.44^***#^
IDH-1 mutation (%)	5 (11.63)	20 (14.81)	17 (17.71)	1 (5.26)	10 (22.22)	7 (30.43)

^*^, ^**^, and ^***^ indicated *vs* SSS 0, p < 0.05, <0.01 and <0.001 respectively. ^#^,^ ##^, and^ ###^ indicated *vs* SSS 1, p < 0.05, <0.01 and <0.001 respectively.

**Table 3:** Univariate and Multivariate analysis of SSS in GBMs

Variables	NO.	Derivation cohort				NO.	Validation cohort			
		Univariate analysis		Multivariate analysis			Univariate analysis		Multivariate analysis	
		HR (95% CI)	p-val	HR (95% CI)	p-val		HR (95% CI)	p-val	HR (95% CI)	p-val
SSS										
0	43	Reference	< 0.001	0.857 (0.747 - 0.983)	0.027	19	Reference	< 0.001	0.783 (0.630 - 0.971)	0.026
1	135	0.649 (0.436 - 0.968)				45	0.659 (0.479 - 0.907)			
2	96	0.747 (0.647 - 0.864)				23	0.699 (0.562 - 0.869)			
**Gender**										
female	111	1.009 (0.750 - 1.358)	0.951	1.014 (0.748 - 1.373)	0.931	40	0.914 (0.534 - 1.536)	0.742	0.914 (0.526 - 1.589)	0.750
male	163	Reference				47	Reference			
**IDH-1 R132H**										
Mutation	42	0.589 (0.383 - 0.907)	0.016	0.595 (0.385 - 0.920)	0.020	17	0.499 (0.243 - 1.025)	0.058	0.397 (0.186 - 0.847)	0.017
Wild-type	232	Reference				70	Reference			
**Resection**										
GTR	189	0.721 (0.532 - 0.978)	0.036	0.763 (0.560 - 1.039)	0.086	56	0.784 (0.444 - 1.383)	0.784	0.714 (0.394 - 1.292)	0.265
non-GTR	85	Reference				31	Reference			
**Chemoradiotherapy**										
Complete	159	0.432 (0.323 - 0.578)	< 0.001	0.444 (0.330 - 0.597)	< 0.001	40	0.268 (0.151 - 0.475)	< 0.001	0.240 (0.131 - 0.441)	< 0.001
Incomplete	115	Reference				47	Reference			

## References

[1] Ostrom QT, Gittleman H, Liao P, Vecchione-Koval T, Wolinsky Y, Kruchko C (2017). CBTRUS Statistical Report: Primary brain and other central nervous system tumors diagnosed in the United States in 2010-2014. Neuro Oncol.

[2] Louis DN, Perry A, Reifenberger G, von Deimling A, Figarella-Branger D, Cavenee WK (2016). The 2016 World Health Organization Classification of Tumors of the Central Nervous System: a summary. Acta neuropathologica.

[3] Bettegowda C, Sausen M, Leary RJ, Kinde I, Wang Y, Agrawal N (2014). Detection of circulating tumor DNA in early- and late-stage human malignancies. Sci Transl Med.

[4] De Mattos-Arruda L, Mayor R, Ng CK, Weigelt B, Martinez-Ricarte F, Torrejon D (2015). Cerebrospinal fluid-derived circulating tumour DNA better represents the genomic alterations of brain tumours than plasma. Nat Commun.

[5] Wang PF, Song HW, Cai HQ, Kong LW, Yao K, Jiang T (2017). Preoperative inflammation markers and IDH mutation status predict glioblastoma patient survival.

[6] Han S, Liu Y, Li Q, Li Z, Hou H, Wu A (2015). Pre-treatment neutrophil-to-lymphocyte ratio is associated with neutrophil and T-cell infiltration and predicts clinical outcome in patients with glioblastoma. BMC Cancer.

[7] Lopes M, Carvalho B, Vaz R, Linhares P (2018). Influence of neutrophil-lymphocyte ratio in prognosis of glioblastoma multiforme. Journal of neuro-oncology.

[8] Xu WZ, Li F, Xu ZK, Chen X, Sun B, Cao JW (2017). Preoperative albumin-to-globulin ratio and prognostic nutrition index predict prognosis for glioblastoma. Onco Targets Ther.

[9] Borg N, Guilfoyle MR, Greenberg DC, Watts C, Thomson S (2011). Serum albumin and survival in glioblastoma multiforme. Journal of neuro-oncology.

[10] He ZQ, Duan H, Ke C, Zhang XH, Guo CC, Al-Nahari F (2017). Evaluation of cumulative prognostic score based on pretreatment plasma fibrinogen and serum albumin levels in patients with newly diagnosed high-grade gliomas. Oncotarget.

[11] Stupp R, Mason WP, van den Bent MJ, Weller M, Fisher B, Taphoorn MJ (2005). Radiotherapy plus concomitant and adjuvant temozolomide for glioblastoma. The New England journal of medicine.

[12] Wang PF, Liu N, Song HW, Yao K, Jiang T, Li SW (2016). IDH-1R132H mutation status in diffuse glioma patients: implications for classification. Oncotarget.

[13] Zhou XW, Dong H, Yang Y, Luo JW, Wang X, Liu YH (2016). Significance of the prognostic nutritional index in patients with glioblastoma: A retrospective study. Clinical neurology and neurosurgery.

[14] Auezova R, Ryskeldiev N, Doskaliyev A, Kuanyshev Y, Zhetpisbaev B, Aldiyarova N (2016). Association of preoperative levels of selected blood inflammatory markers with prognosis in gliomas. Onco Targets Ther.

[15] Xu W, Wang D, Zheng X, Ou Q, Huang L (2017). Sex-dependent association of preoperative hematologic markers with glioma grade and progression.

[16] Galizia G, Lieto E, Auricchio A, Cardella F, Mabilia A, Podzemny V (2017). Naples Prognostic Score, Based on Nutritional and Inflammatory Status, is an Independent Predictor of Long-term Outcome in Patients Undergoing Surgery for Colorectal Cancer. Dis Colon Rectum.

[17] Yan H, Parsons DW, Jin G, McLendon R, Rasheed BA, Yuan W (2009). IDH1 and IDH2 mutations in gliomas. The New England journal of medicine.

[18] Ikemura M, Shibahara J, Mukasa A, Takayanagi S, Aihara K, Saito N (2016). Utility of ATRX immunohistochemistry in diagnosis of adult diffuse gliomas. Histopathology.

[19] Bambury RM, Teo MY, Power DG, Yusuf A, Murray S, Battley JE (2013). The association of pre-treatment neutrophil to lymphocyte ratio with overall survival in patients with glioblastoma multiforme. Journal of neuro-oncology.

[20] Massara M, Persico P, Bonavita O, Mollica Poeta V, Locati M, Simonelli M (2017). Neutrophils in Gliomas. Front Immunol.

[21] Menter DG, Kopetz S, Hawk E, Sood AK, Loree JM, Gresele P (2017). Platelet "first responders" in wound response, cancer, and metastasis. Cancer Metastasis Rev.

[22] Kmiecik J, Poli A, Brons NH, Waha A, Eide GE, Enger PO (2013). Elevated CD3+ and CD8+ tumor-infiltrating immune cells correlate with prolonged survival in glioblastoma patients despite integrated immunosuppressive mechanisms in the tumor microenvironment and at the systemic level. J Neuroimmunol.

[23] Gieryng A, Pszczolkowska D, Walentynowicz KA, Rajan WD, Kaminska B (2017). Immune microenvironment of gliomas. Lab Invest.

[24] Chojkier M (2005). Inhibition of albumin synthesis in chronic diseases: molecular mechanisms. J Clin Gastroenterol.

[25] Nduom EK, Weller M, Heimberger AB (2015). Immunosuppressive mechanisms in glioblastoma. Neuro Oncol.

[26] Niwa N, Matsumoto K, Ide H, Nagata H, Oya M (2018). Prognostic Value of Pretreatment Albumin-to-Globulin Ratio in Patients With Non-Muscle-Invasive Bladder Cancer.

[27] Zitvogel L, Pietrocola F, Kroemer G (2017). Nutrition, inflammation and cancer. Nat Immunol.

[28] Siegel EM, Nabors LB, Thompson RC, Olson JJ, Browning JE, Madden MH (2013). Prediagnostic body weight and survival in high grade glioma. Journal of neuro-oncology.

[29] Liang R, Li J, Li M, Yang Y, Wang X, Mao Q (2017). Clinical significance of pre-surgical serum lipid levels in patients with glioblastoma. Oncotarget.

[30] Steinbrecher KA, Horowitz NA, Blevins EA, Barney KA, Shaw MA, Harmel-Laws E (2010). Colitis-associated cancer is dependent on the interplay between the hemostatic and inflammatory systems and supported by integrin alpha(M)beta(2) engagement of fibrinogen. Cancer Res.

[31] Degen JL, Palumbo JS (2012). Hemostatic factors, innate immunity and malignancy. Thromb Res.

[32] Zadora P, Dabrowski W, Czarko K, Smolen A, Kotlinska-Hasiec E, Wiorkowski K (2015). Preoperative neutrophil-lymphocyte count ratio helps predict the grade of glial tumor - a pilot study. Neurol Neurochir Pol.

[33] Zheng SH, Huang JL, Chen M, Wang BL, Ou QS, Huang SY (2017). Diagnostic value of preoperative inflammatory markers in patients with glioma: a multicenter cohort study.

[34] Mason M, Maurice C, McNamara MG, Tieu MT, Lwin Z, Millar BA (2017). Neutrophil-lymphocyte ratio dynamics during concurrent chemo-radiotherapy for glioblastoma is an independent predictor for overall survival. Journal of neuro-oncology.

[35] Moschetta M, Uccello M, Kasenda B, Mak G, McClelland A, Boussios S (2017). Dynamics of Neutrophils-to-Lymphocyte Ratio Predict Outcomes of PD-1/PD-L1 Blockade. BioMed research international.

